# TFAM-deficient mouse skin fibroblasts – an *ex vivo* model of mitochondrial dysfunction

**DOI:** 10.1242/dmm.048995

**Published:** 2021-08-25

**Authors:** Manuel J. Del Rey, Carolina Meroño, Cristina Municio, Alicia Usategui, María Mittelbrunn, Inés García-Consuegra, Gabriel Criado, José L. Pablos

**Affiliations:** 1 Grupo de Enfermedades Inflamatorias y Autoinmunes, Instituto de Investigación Hospital 12 de Octubre (i+12), 28041 Madrid, Spain; 2 Departamento de Biología Molecular, Centro de Biología Molecular Severo Ochoa (CBMSO), Consejo Superior de Investigaciones Científicas (CSIC)-Universidad Autónoma de Madrid (UAM), 28049 Madrid, Spain; 3 Instituto de Investigación Hospital 12 de Octubre (i+12), 28041 Madrid, Spain; 4 Unidad de Proteómica, Instituto de Investigación Hospital 12 de Octubre (i+12), 28041 Madrid, Spain; 5 Centro de Investigación Biomédica en Red de Enfermedades Raras (CIBERER), 28029 Madrid, Spain; 6 Departamento de Medicina, Universidad Complutense de Madrid, 28040 Madrid, Spain

**Keywords:** TFAM, Mitochondrial dysfunction, Fibroblasts, Inflammation, Cellular senescence

## Abstract

Mitochondrial dysfunction associates with several pathological processes and contributes to chronic inflammatory and ageing-related diseases. Mitochondrial transcription factor A (TFAM) plays a critical role in maintaining mtDNA integrity and function. Taking advantage of *Tfam*^*fl/fl*^ UBC-Cre/ER^T2+/+^ mice to investigate mitochondrial dysfunction in the stromal cell component, we describe an inducible *in vitro* model of mitochondrial dysfunction by stable depletion of TFAM in primary mouse skin fibroblasts (SK-FBs) after 4-hydroxytamoxifen (4-OHT) administration. *Tfam* gene deletion caused a sustained reduction in *Tfam* and mtDNA-encoded mRNA in Cre(+) SK-FBs cultured for low (LP) and high (HP) passages that translated into a loss of TFAM protein. TFAM depletion led to a substantial reduction in mitochondrial respiratory chain complexes that was exacerbated in HP SK-FB cultures. The assembly pattern showed that the respiratory complexes fail to reach the respirasome in 4-OHT-treated Cre(+) SK-FBs. Functionally, mito-stress and glycolysis-stress tests showed that mitochondrial dysfunction developed after long-term 4-OHT treatment in HP Cre(+) SK-FBs and was compensated by an increase in the glycolytic capacity. Finally, expression analysis revealed that 4-OHT-treated HP Cre(+) SK-FBs showed a senescent and pro-inflammatory phenotype.

## Abstract (precis)

**Summary:** TFAM depletion in skin fibroblasts *ex vivo* results in a senescent and inflammatory phenotype, providing a valuable model to investigate the role of mitochondrial dysfunction in ageing and inflammatory pathologies.

## INTRODUCTION

Mitochondria are important organelles, critical to the generation of cellular energy and synthesis of biomolecules, contributing to the regulation of metabolic homeostasis and cell adaptation to stress. Mitochondria contain the necessary machinery to carry out different processes of energy production, such as oxidative phosphorylation (OXPHOS), the tricarboxylic acid cycle or fatty acid oxidation, as well as the biosynthesis of amino acids, lipids and nucleotides, and the maintenance of homeostatic levels of calcium (Murphy and Hartley, 2018).

Altered mitochondrial function is linked to different pathological conditions, such as metabolic and neurodegenerative disorders, cancer, heart failure and ischemia-reperfusion injury. Mitochondrial dysfunction plays a central role in acute and chronic inflammatory diseases, particularly in ageing-related inflammatory and degenerative diseases (López-Armada et al., 2013; Murphy and Hartley, 2018). In particular, metabolic changes in the immune system have been associated with age-related deterioration in different tissues, leading to multimorbidity and premature death (Desdín-Micó et al., 2020). In damaged mitochondria, there is a disruption of ATP synthesis and a production of reactive oxygen species (ROS) that results in activation of the NF-kB pathway and the release of inflammatory factors, similar to the senescence-associated secretory phenotype (SASP) (Wiley et al., 2016). Another way by which mitochondria contribute to inflammation is through mitochondrial DNA (mtDNA) hypomethylation, a pattern that can act as a damage-associated molecular pattern (DAMP), triggering an immune response when released into the cytoplasm due to a variety of cellular stresses (Riley and Tait, 2020).

Several observations support a role for mitochondrial dysfunction of the stromal cell component in the onset or progression of inflammatory diseases. It has been reported that impaired mitochondrial activity increases inflammatory responsiveness to cytokines in normal human chondrocytes (Vaamonde-García et al., 2012). Mutations in mtDNA due to hypoxia and inflammatory conditions in synovial tissues promote mitochondrial dysfunction and potentiate the pro-inflammatory response of synovial fibroblasts (Biniecka et al., 2016; Harty et al., 2012; Valcárcel-Ares et al., 2014). Similarly, mitochondrial dysfunction in cancer-associated fibroblasts has been associated with an increase in their aggressive behaviour (Balliet et al., 2011).

Mitochondria contain their own genome encoding for 13 electron-transport-chain proteins, core constituents of the respiratory complexes, and specific 22 transfer RNAs (tRNAs) and two ribosomal RNAs (rRNAs). However, different nucleus-encoded proteins are also essential for mitochondrial function and mtDNA maintenance, with mitochondrial transcription factor A (TFAM) being the most abundant mtDNA-associated protein (Friedman and Nunnari, 2014). TFAM is essential for the regulation of mtDNA transcription and replication, and prevents it from being naked and exposed to degradation (Alam et al., 2003; Kang et al., 2007; Larsson et al., 1998). Thus, TFAM deficiency may lead to mitochondrial stress and mtDNA release (Riley and Tait, 2020).

Larsson et al. (1998) described a *Tfam* knockout (KO) mouse model that showed the essential nature of TFAM, because homozygous KO (*Tfam^−/−^*) embryos died prior to embryonic day 10.5. However, heterozygous *Tfam* KO (*Tfam^+/−^*) mice were viable and showed a reduction in mtDNA copy number and respiratory chain malfunction, indicating that *Tfam* depletion could be used to create a valid model to study mitochondrial dysfunction (Larsson et al., 1998). Alternatively, cell-type-specific *Tfam* KO mice have shown deleterious effects in cardiomyocytes (Li et al., 2000), pancreatic β-cells (Silva et al., 2000) or skeletal muscle (Wredenberg et al., 2002). *Tfam* KO in T cells causes lysosomal dysfunction and an exaggerated inflammatory response that induced systemic senescence in peripheral tissues leading to premature age-associated multimorbidity (Baixauli et al., 2015; Desdín-Micó et al., 2020). Interestingly, a similar approach targeting adipocytes has demonstrated both beneficial and detrimental effects depending on the penetrance and distribution of TFAM depletion (Vernochet et al., 2012, 2014).

Fibroblasts are the most abundant cells in the stroma of most tissues, providing mechanical strength for tissues by producing extracellular matrix components. Fibroblasts are critical effectors of inflammation in a variety of pathological conditions such as chronic arthritis and cancer (Barone et al., 2012). Their phenotypic pro-inflammatory changes have been associated with oxidative processes and impaired mitochondrial function (Castejón-Vega et al., 2021; Jędrak et al., 2018). We have generated a cellular inducible model in *Tfam^fl/fl^* UBC-Cre/ER^T2+/+^ skin fibroblasts (SK-FBs) by *ex vivo* treatment with 4-hydroxytamoxifen (4-OHT). This model provides a valuable tool for TFAM targeting in a cell-specific manner and reveals the contribution of mitochondrial dysfunction to the pro-inflammatory phenotype of fibroblasts.

## RESULTS

### Inducible TFAM depletion in primary SK-FBs by 4-OHT

**Fig. 1.**
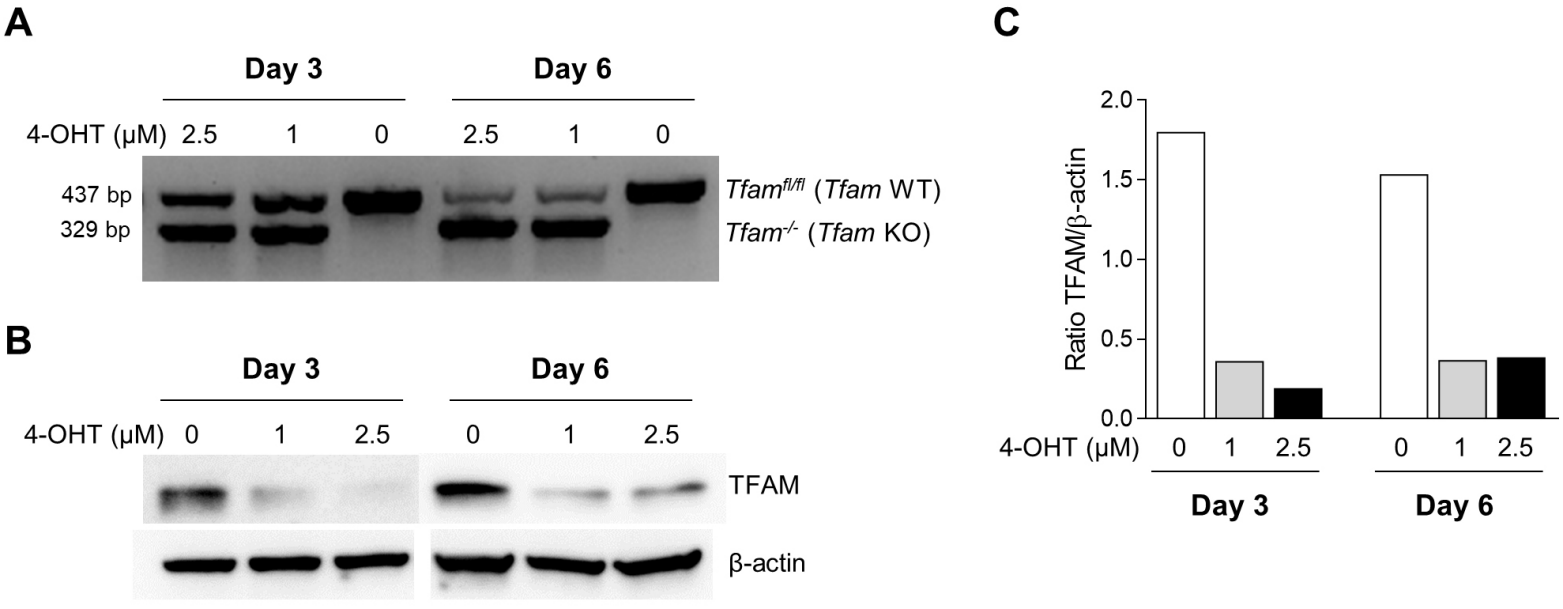
**Dose response and kinetics of 4-OHT treatment on *Tfam* genotype and TFAM protein expression.** Cre(+) SK-FBs were treated with the indicated doses of 4-OHT for 3 or 6 days. (A) PCR analysis of *Tfam* genotype. (B) Representative western blot of TFAM and β-actin protein in a Cre(+) cell line. (C) Densitometric analysis of TFAM protein expression.

The presence or absence of *Cre* gene was confirmed by conventional PCR in *Tfam^fl/fl^* UBC-Cre/ER^T2+/+^ [Cre(+)] and *Tfam^fl/fl^* UBC-Cre/ER^T2−/−^ [Cre(−)] SK-FBs (Fig. S1). To induce deletion of the *Tfam* gene, Cre(+) SK-FBs were treated with 1 µM or 2.5 µM 4-OHT for 3 or 6 days. *Tfam* deletion was time dependent, and a reduction in wild-type (WT) *Tfam* amplification was observed in 4-OHT-treated cells after 3 days, whereas the *Tfam* KO allele was only detected in treated cells. At day 6, *Tfam* WT amplification was further reduced in treated cells and no changes were detected in untreated cells (Fig. 1A). The partial loss of the *Tfam* WT allele in Cre(+) SK-FBs after 4-OHT treatment translated into a more than 75% reduction in TFAM protein levels, regardless of dose and time (Fig. 1B,C).

Based on these results, we defined a treatment strategy to set up an *ex vivo* model of SK-FBs with stable depletion of TFAM (Fig. S2). SK-FBs were treated with an initial dose of 2.5 µM 4-OHT, followed by weekly additions of 1 µM 4-OHT to maintain the effect. Culture medium was supplemented with 50 μg/ml uridine and 1 mM sodium pyruvate after the first 4-OHT administration, and cells were maintained for a low (LP; one to three) or high (HP; four to seven) number of passages to study the impact of *Tfam* gene deletion on the development of mitochondrial dysfunction in SK-FBs. The proliferative capability of SK-FBs was not affected by TFAM deficiency under these culture conditions (Fig. S3).

### Sustained reduction in the expression of mtDNA-encoded genes in TFAM-deficient SK-FBs

**Fig. 2.**
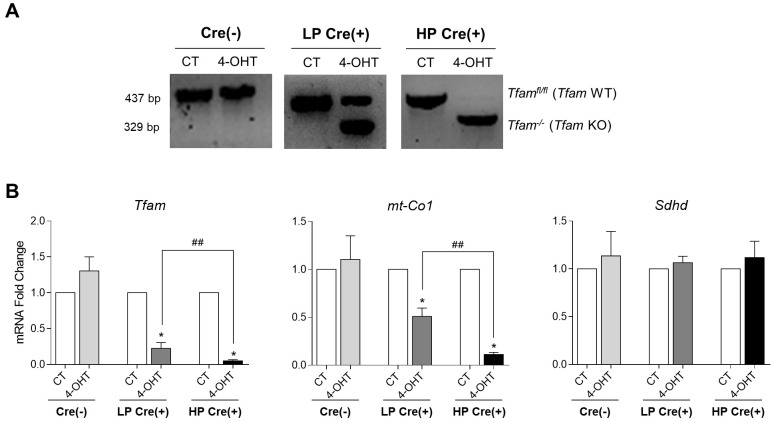
**Expression of *Tfam* and mitochondrial-related genes.** (A) PCR analysis of *Tfam* genotype in treated (4-OHT) or untreated (CT) Cre(+) SK-FBs cultured for low (LP) or high (HP) passages and Cre(−) SK-FBs. [Cre(−) SK-FBs are pooled LP and HP because we found no differences between them.] (B) qRT-PCR analysis of *Tfam*, *mt-Co1* and *Sdhd* transcript levels in Cre(−) cells (*n*=6) and LP or HP Cre(+) cells (*n*=7) (mean±s.e.m.) (*P<0.05, Wilcoxon test; ^##^*P*<0.01, Mann–Whitney test).

*Tfam* genotyping confirmed *Tfam* gene deletion in SK-FBs after long-term culture. Amplification of *Tfam* KO was only detected in LP Cre(+) SK-FB cultures treated with 4-OHT, indicating a shift from *Tfam* WT to *Tfam* KO. This shift was completed in HP cultures, showing a total loss of *Tfam* WT alleles. In contrast, only the *Tfam* WT allele was detected in untreated control (CT) Cre(+), CT Cre(−) and 4-OHT-treated Cre(−) SK-FBs, regardless of the passage (Fig. 2A).

As expected, *Tfam* transcript levels in LP Cre(+) SK-FBs displayed a significant reduction (*P*=0.016) in 4-OHT-treated cells compared to CT cells that was sustained in HP cultures (*P*=0.016), whereas no significant differences were observed between 4-OHT-treated and CT Cre(−) cells. *Tfam* expression was also significantly lower in HP compared to LP 4-OHT-treated Cre(+) SK-FBs (fold change values 0.051 versus 0.224, *P*=0.008) (Fig. 2B).

TFAM is crucial for the regulation of the mtDNA transcription and prevents its degradation; therefore, it is important to determine whether the loss of TFAM had a deleterious effect on mtDNA without affecting nuclear DNA. Mitochondrial DNA damage was evaluated by the expression of mtDNA-encoded cytochrome C oxidase subunit 1 (*mt-Co1*) mRNA, a component of the mitochondrial respiratory chain (MRC). We observed a significant reduction in *mt-Co1* expression (*P*=0.031) in 4-OHT-treated compared to CT LP Cre(+) SK-FBs, that was again sustained in treated HP Cre(+) SK-FBs (*P*=0.031), whereas no difference was observed between 4-OHT-treated and CT Cre(−) cells. Similar to *Tfam* expression, a significant reduction in *mt-Co1* expression was found in 4-OHT-treated HP compared to LP Cre(+) cells (fold change values 0.111 versus 0.509, *P*=0.002) (Fig. 2B).

Succinate dehydrogenase cytochrome b small subunit (*Sdhd*) was used as a control of nuclear expression of mitochondrial genes. *Sdhd* mRNA expression was not altered by the 4-OHT treatment or by the number of passages (Fig. 2B).

### Expression of TFAM and mtDNA-encoded proteins is abrogated in TFAM-deficient SK-FBs

**Fig. 3.**
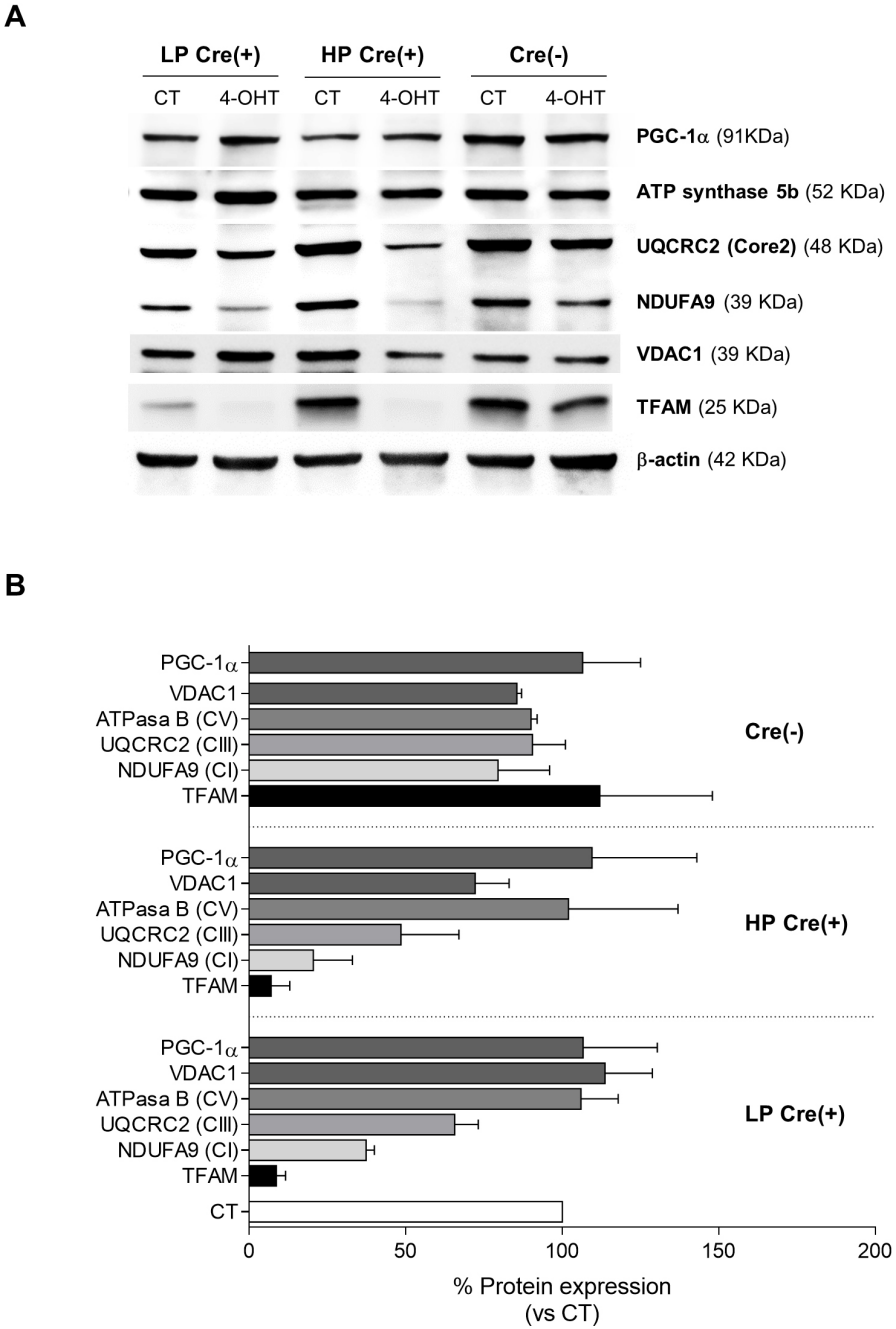
**Expression of TFAM and mitochondrial proteins.** (A) Representative western blots of TFAM, PGC-1α, ATP synthase subunit 5b, VDAC1, UQCRC2 (core protein 2) and NDUFA9 proteins in LP or HP Cre(+) and Cre(−) cell lines. (B) Densitometric quantification of protein expression, shown as the percentage of CT cells for each cell line. Cre(−) (*n*=2) and LP and HP Cre(+) (*n*=3) were used (mean±s.e.m.).

We performed a series of western blot analyses to investigate the effect of *Tfam* gene knockout on TFAM protein, and its implications on mitochondrial content and mtDNA-encoded proteins. First, we confirmed TFAM protein depletion in 4-OHT-treated Cre(+) SK-FBs, whereas no effect was observed in CT cells (Fig. 3). TFAM expression was detected similarly in 4-OHT-treated and CT Cre(−) cells, but was reduced by more than 90% in LP and HP 4-OHT-treated Cre(+) SK-FBs (Fig. 3B).

Next, we quantified the levels of PGC-1α (PPARGC1A), ATP synthase and VDAC1 to address the impact of TFAM depletion on mitochondrial biogenesis and mass (Feichtinger et al., 2019; Knutti and Kralli, 2001; Scarpulla, 2008), but detected no significant changes in the expression of these proteins in 4-OHT-treated LP and HP Cre(+) SK-FBs compared to CT cells (Fig. 3B). As expected, TFAM depletion led to a reduction in proteins that are integral parts of the MRC complexes; specifically, lower expression of NDUFA9 (CI subunit) and UQCRC2 Core2 (CIII subunit) was observed (Fig. 3A,B). This reduction was more pronounced in HP compared to LP Cre(+) cells (79.5% versus 64.7% reduction in CI and 51.5% versus 36.3% reduction in CIII). These findings suggest that TFAM does not play a role in mitochondrial biogenesis or maintaining the mitochondrial mass, and that TFAM depletion critically affects the levels of MRC.

### Reduction in respiratory chain supercomplexes and the respirasome in TFAM-deficient SK-FBs

MRC enzymes are associated into complexes and supercomplexes (SCs) that are structurally interdependent and assemble plastically to accomplish a maximal-efficient electron transfer (Lobo-Jarne and Ugalde, 2018). To explore the effect of TFAM depletion on MRC assembly, we performed blue native electrophoresis (BNE) in HP Cre(+) SK-FBs.

**Fig. 4.**
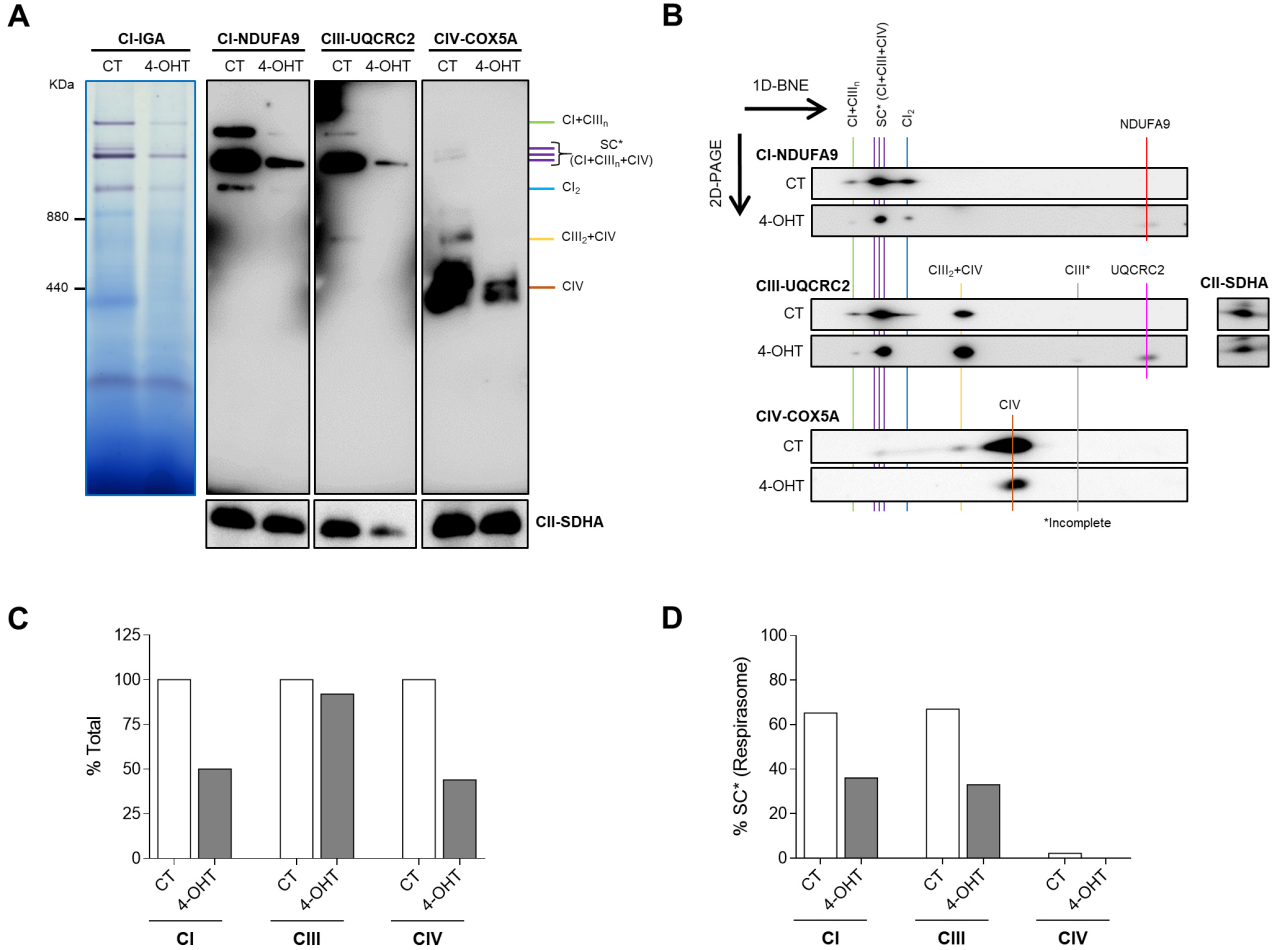
**Mitochondrial supercomplexes (SCs) in SK-FBs.** (A) Representative images of a complex I in-gel activity (CI-IGA) assay and a one-dimensional blue native electrophoresis (1D-BNE) analysis of digitonin-solubilized mitochondria from control (CT) and treated (4-OHT) HP Cre(+) SK-FBs. 1D-BNE shows OXPHOS complexes I to IV, using complex II (CII-SDHA) as a loading control. (B) Two-dimensional-polyacrylamide gel electrophoresis (2D-PAGE) analysis of the steady-state levels of respirasome and different intermediate SCs. Images show a representative line of treated (4-OHT) or untreated (CT) HP Cre(+) SK-FBs. (C,D) Densitometric quantification of total complex I, III and IV (C), and the fraction of each complex contributing to the respirasome (SC*) (D), shown as the percentage of the CT.

Complex I in-gel activity assay (CI-IGA) and anti-NDUFA9 (CI subunit) revealed much lower levels of the complexes containing SCs (i.e. SC I+III_n_ and respirasome SC* I+III_n_+IV) in Cre(+) SK-FBs treated with 4-OHT, compared to control cells (Fig. 4A). Anti-UQCRC2 (CIII subunit) and anti-COX5A (CIV subunit) immunoblotting confirmed the lower amounts of respirasome SC* and SC I+III_n_ in parallel with reduced levels of SC III_2_+IV and free CIV in 4-OHT-treated cells (Fig. 4A).

Two-dimensional (2D)-polyacrylamide gel electrophoresis (PAGE) analysis revealed alterations in the assembly pattern of the complexes and supercomplexes in 4-OHT-treated compared to CT SK-FBs (Fig. 4B). Quantitatively, TFAM KO SK-FBs showed lower levels of CI and CIV (reduction by 50%) and similar levels of CIII in absolute terms, regardless of the assembly (Fig. 4C). In contrast, analysis of the respirasome, the functional supercomplex, showed that CIV failed to reach the respirasome and that expression of both CI and CIII was reduced by 50% in 4-OHT SK-FBs (Fig. 4D). The presence of incomplete CIII and free NDUFA9 and UQCRC2 enzymes was also detected in 4-OHT-treated cells (Fig. 4B).

### Long-term TFAM depletion causes mitochondrial dysfunction

**Fig. 5.**
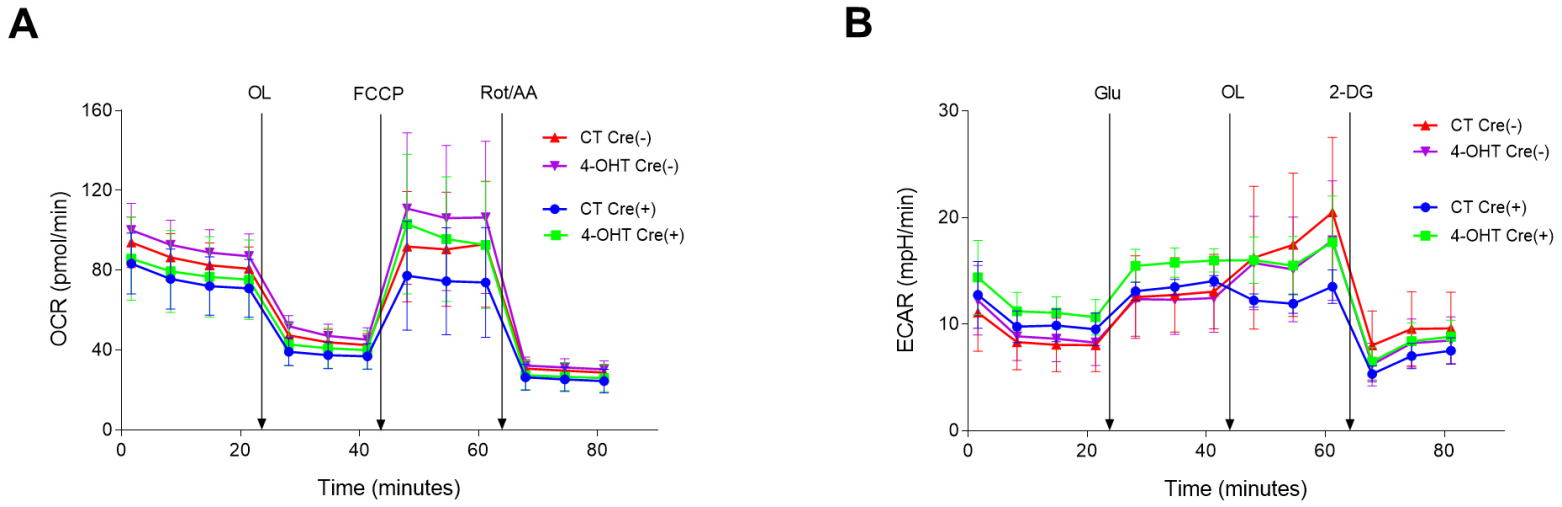
**Mitochondrial function and glycolytic activity in LP SK-FBs.** (A) Oxygen consumption rate (OCR) in mito-stress test, basal and after sequential injections of oligomycin (OL), carbonyl cyanide-p-trifluoromethoxyphenylhydrazone (FCCP) and rotenone/antimycin A (Rot/AA). (B) Extracellular acidification rate (ECAR) in the glycolysis-stress test, basal and after sequential injections of glucose (Glu), OL and 2-deoxy D-glucose (2-DG). Treated (4-OHT) and untreated (CT) Cre(−) (*n*=3) and Cre(+) (*n*=3) cells are shown (mean±s.e.m.).

To characterize the functional effects of the mitochondrial damage caused by *Tfam* gene depletion, we assessed mitochondrial function and glycolytic activity. Under conditions of partial TFAM depletion, i.e. LP cell cultures, oxygen consumption rate (OCR) levels in a mito-stress test were comparable between 4-OHT-treated and CT Cre(+) and Cre(−) SK-FBs (Fig. 5A). Likewise, extracellular acidification rate (ECAR) following a glycolysis-stress test showed no differences between the experimental groups (Fig. 5B).

**Fig. 6.**
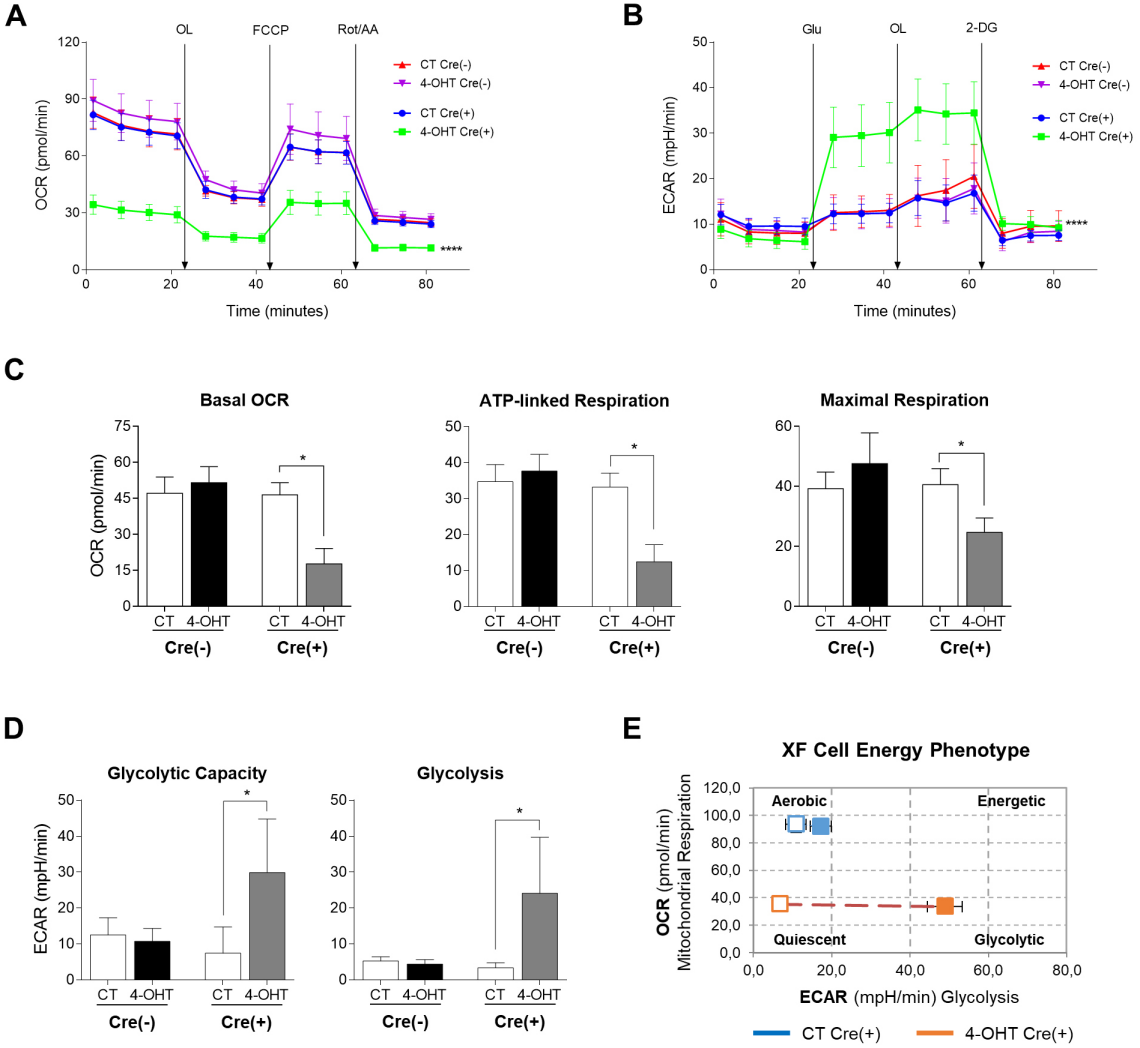
**Mitochondrial function and glycolytic activity in HP SK-FBs.** (A) OCR in mito-stress test, basal and after consecutive injections of OL, FCCP and Rot/AA (****P<0.0001, two-way ANOVA with Tukey's multiple comparison test). (B) ECAR in glycolysis-stress test, basal and after sequential injections of Glu, OL and 2-DG (****P<0.0001, two-way ANOVA with Tukey's multiple comparison test). (C) Quantification of basal OCR, ATP-linked respiration and maximal respiration (*P<0.05, Wilcoxon test). (D) Quantification of glycolytic capacity and glycolysis (*P<0.05, Wilcoxon test). (E) Representation of the XF cell energy phenotype. Treated (4-OHT) and untreated (CT) Cre(−) (*n*=3) and Cre(+) (*n*=3) SK-FBs are shown (mean±s.e.m.).

Mito-stress test in HP cultures with sustained TFAM depletion and mtDNA damage showed a significant reduction in OCR throughout the assay in 4-OHT-treated Cre(+) compared to CT Cre(+) and Cre(−) SK-FBs (Fig. 6A, *P*<0.0001). Basal OCR and, consequently, ATP-linked respiration, considered as the basal respiratory portion used to produce ATP according to the effect of the ATP synthase inhibition by oligomycin, were significantly decreased in treated Cre(+) SK-FBs (Fig. 6C, *P*=0.03 for both comparisons). In addition, maximal respiration was also significantly decreased in 4-OHT-treated Cre(+) cells (Fig. 6C, *P*=0.03). Glycolysis-stress test in 4-OHT-treated Cre(+) SK-FBs showed an exaggerated ECAR increase in response to glucose injection compared to CT Cre(+) and Cre(−) SK-FBs (Fig. 6B, *P*<0.0001). The high ECAR observed in TFAM-depleted cells was maintained upon ATP synthase inhibition by oligomycin, causing a significant increase in glycolytic capacity (*P*=0.03) in comparison to CT Cre(+) SK-FBs (Fig. 6D). Fig. 6E provides an illustrative example of the different cell energy phenotypes caused by TFAM depletion; the graphic reflects the aerobic phenotype of CT Cre(+) SK-FBs and the phenotype switch from quiescent to glycolytic that 4-OHT-treated Cre(+) SK-FBs undergo after glucose injection.

All parameters obtained after mito-stress and glycolytic assays confirmed that mitochondrial dysfunction was achieved after long-term 4-OHT treatment in HP Cre(+) SK-FBs and the ensuing extended damage to mtDNA.

### TFAM-deficient SK-FBs display a senescent pro-inflammatory phenotype

Fibroblasts play a physiological role in connective tissue homeostasis and repair through the production of extracellular matrix proteins. To analyse the potential impact of mitochondrial dysfunction on this process we evaluated the gene expression of relevant matrix components in Cre(+) SK-FBs. No differences in the expression of *Col1a1*, *Fn1* and *Acta2* were observed in TFAM-depleted SK-FBs compared to CT SK-FBs (Fig. S4A). Immunofluorescent detection confirmed that levels of alpha-smooth muscle actin (α-SMA; ACTA2) were not affected by mitochondrial dysfunction (Fig. S4B).

In addition to its role in metabolism, mitochondrial dysfunction is also involved in the development of cellular senescence and inflammatory responses. To address the potential contribution of TFAM depletion to these processes in SK-FBs, we analysed the expression of cell senescence and inflammatory markers.

**Fig. 7.**
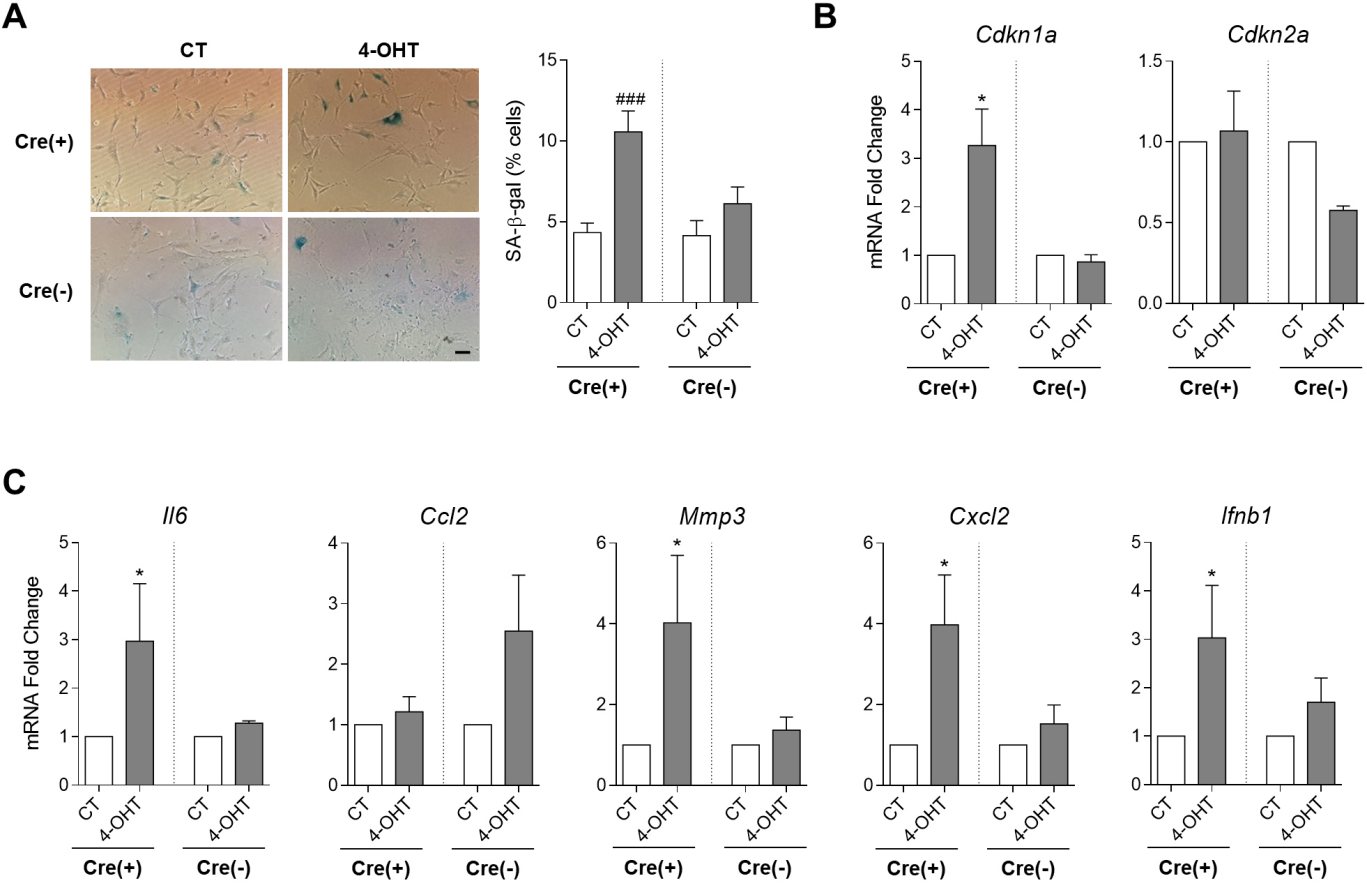
**Analysis of senescent and inflammatory markers in TFAM KO SK-FBs.** (A) Colorimetric detection of senescence-associated beta-galactosidase (SA-β-gal) activity (left) and quantification of SA-β-gal(+) treated (4-OHT) and untreated (CT) Cre(−) (*n*=2) and Cre(+) (*n*=4) SK-FBs (right) (^###^*P*<0.001, Mann–Whitney test). Scale bar: 200 μm. (B,C) Changes in mRNA expression of senescent markers *Cdkn1a* and *Cdkn2a* (B) and inflammatory mediators *Il6*, *Ccl2*, *Mmp3*, *Cxcl2* and *Ifnb1* (C) in 4-OHT HP Cre(+) (*n*=9) and Cre(−) (*n*=4) cells with respect to CT cells (mean±s.e.m.) (*P<0.05, Wilcoxon test).

4-OHT treatment resulted in a significant increase in senescence-associated beta-galactosidase (SA-β-gal)(+) cells in Cre(+) SK-FB cultures (*P*=0.0004) (Fig. 7A). Likewise, expression of the p21-encoding gene (*Cdkn1a*) was significantly increased in 4-OHT-treated SK-FBs, whereas no changes were observed in the expression of the p16-encoding gene (*Cdkn2a*) (Fig. 7B). We have previously shown that premature senescence of primary synovial fibroblasts is associated with the so-called SASP that results in an enhanced inflammatory response (Del Rey et al., 2019). Thus, we explored whether this phenotype is reproduced after the mitochondrial dysfunction triggered by TFAM depletion in SK-FBs.

Analysis of SASP components showed a significant upregulation of the expression of the inflammatory mediators *Il6* (*P*=0.027), *Mmp3* (*P*=0.008), *Cxcl2* (*P*=0.039) and *Ifnb1* (*P*=0.019) in 4-OHT-treated Cre(+) SK-FBs, but no changes were observed in the chemokine *Ccl2* (Fig. 7C). This upregulation did not seem to be related to alteration of NF-κB or MAPK signalling because basal phosphorylation of p65 and p38 proteins was not affected by TFAM depletion (data not shown).

Collectively, these results indicate that mitochondrial dysfunction promoted an increase in cellular senescence and a pro-inflammatory phenotype in SK-FBs.

## DISCUSSION

Mitochondria are essential organelles for energy metabolism and cellular homeostasis. Impaired mitochondrial function has been associated with a wide variety of pathological processes, with the involvement of different cell types, including both immune and stromal cells (Annesley and Fisher, 2019; Ghesquière et al., 2014). In recent years, metabolism has been the subject of multiple investigations exploring therapeutic targets against these pathologies (McGarry and Fearon, 2019; Mehta and Chandel, 2015; Murphy and Hartley, 2018). Here, we describe the generation and validation of an inducible model of mitochondrial dysfunction in SK-FBs from *Tfam^fl/fl^* UBC-Cre/ER^T2+/+^ mice that provides a valuable tool to investigate these processes.

*Tfam* KO induction was achieved in SK-FB cultures from *Tfam* floxed mice containing the *Cre* gene after 4-OHT treatment. The use of 4-OHT over tamoxifen is preferable because of its higher affinity and reduced toxicity, an important parameter to take into account when analysing long-term biological processes. In this context, it is also relevant to consider that 4-OHT itself has been found to have an effect on mitochondria by acting as an inhibitor of the mitochondrial permeability transition, thus preventing the development of mitochondrial inflammation and consequent cell death, and providing protection against the Ca^2+^-induced mitochondrial swelling, among other effects (Cardoso et al., 2002). To circumvent this potential protective role of 4-OHT in mitochondria, we included control groups of 4-OHT-treated Cre(−) cells in our experiments and found that their functional responses were not affected.

4-OHT treatment led to a shift in *Tfam* WT towards *Tfam* KO genotype that in turn led to a decrease in *Tfam* transcripts and loss of TFAM protein, inducing a reduction in mtDNA level, while nuclear DNA gene transcription remained intact. These events were maintained after long-term culture under these conditions and the viability of the cells was not reduced, allowing the establishment of a stable *Tfam*-depleted cell culture.

The analysis of this process at different culturing times, which we classified as LP or HP, showed interesting findings. Initially, there is a partial shift in *Tfam* WT towards *Tfam* KO genotype in LP SK-FBs. Although *Tfam* gene expression was already reduced dramatically in these LP cells, a significant difference was generated between 4-OHT-treated LP and HP Cre(+) cells in terms of mtDNA transcripts, as their expression was further reduced when the cells were maintained in culture for longer time. These results are in agreement with previous reports from de Oliveira et al. (2020) based on CRISPR-edited TFAM-deficient bovine fibroblasts, in which they observed an early reduction in mtDNA that was further increased in late passages. This indicates a cumulative mtDNA damage that would lead to a decrease in the production of mtDNA-encoded proteins such as MRC complex subunits as described (Silva and Larsson, 2002). In our model, the fast decrease in *Tfam* transcript and protein levels in LP cells is not accompanied by an accelerated decrease in mtDNA, the reduction in which is more progressive. This fact indicates that the protective action of TFAM on mtDNA is very stable, and therefore only the sustained absence of the protein is capable of exposing mtDNA for degradation.

The modest reduction in mtDNA levels of LP SK-FBs was also translated into a reduction in MRC expression levels that nonetheless allowed the maintenance of an intact mitochondrial function. According to England et al. (1978), the functional effect caused by *Tfam* KO in mitochondria exhibits a time lag since its disruption, presumably due to the higher stability of mtDNA-encoded transcripts and proteins as a response to the inhibition of mtDNA expression. Instead, SK-FBs maintained for a longer time in culture (HP) showed a heightened mtDNA damage that led to an altered metabolism characterized by a reduction in the basal OCR and a very low maximal respiration rate, suggesting mitochondrial dysfunction. The impaired mitochondrial function resulted in decreased ATP production that is compensated by increasing the glycolytic activity to obtain energy. HP TFAM-depleted cells maintain the respiratory function despite the drastic reduction in OCRs promoted by mitochondrial dysfunction, as illustrated by the presence, albeit at lower levels than control cells, of assembled complexes in the respirasome, the main functional unit of the OXPHOS system (Lobo-Jarne and Ugalde, 2018).

In summary, TFAM KO SK-FBs undergo a shift from OXPHOS to glycolysis due to the mitochondrial damage caused by TFAM depletion that prevents them from having an efficient OXPHOS. Our model displays a phenomenon similar to the Warburg effect, first described in cancer cells and extended later to activated immune cells and other cell types, including fibroblasts, in different pathological conditions (Garcia-Carbonell et al., 2016; Krawczyk et al., 2010; O'Neill and Pearce, 2016; Vincent et al., 2008; Warburg, 1956). In relation to TFAM depletion, our results are in the line with previous reports that show decreased OCR, increased ECAR and glycolytic reprogramming in TFAM-depleted T cells (Baixauli et al., 2015; Desdín-Micó et al., 2020).

A prominent role of fibroblasts in the generation and persistence of tissue inflammation has been recognized in recent years. Several reports have highlighted the association of impaired mitochondrial function in fibroblasts with a pathological phenotype, i.e. SK-FBs in systemic sclerosis (Luckhardt and Thannickal, 2015) and Huntington's disease (Jędrak et al., 2018), synovial fibroblasts in rheumatoid arthritis (Bartok and Firestein, 2010) and stromal fibroblasts from the tumour microenvironment promoting breast cancer tumour growth (Balliet et al., 2011). SK-FBs with depleted TFAM exhibited a pro-inflammatory phenotype, thus providing a suitable mitochondrial dysfunction model for the better understanding of fibroblast-related inflammatory pathologies as well as for the design and testing of therapeutic strategies. Alternatively, the *in vitro* model described here can be adapted to achieve specific TFAM depletion in other cell types affected by mitochondrial dysfunction in a variety of disorders.

Because the association between ageing, cell senescence and mitochondrial dysfunction is widely known (Birch and Passos, 2017; Vizioli et al., 2020; Wiley et al., 2016), another application of the model characterized in this work could be in the field of cellular senescence and ageing-related pathologies. Recently, it was described that *Tfam*-depleted T cells are able to induce systemic senescence and contribute to premature ageing in peripheral tissues by the release of inflammatory mediators (Desdín-Micó et al., 2020). Our results have also demonstrated an increased senescence in TFAM KO SK-FB cultures accompanied by mRNA overexpression of SASP mediators such as *Il6*, *Mmp3* or *Cxcl2*.

In conclusion, our data describe a robust cell-type-specific *ex vivo* model of mitochondrial dysfunction induced by 4-OHT in fibroblasts that results in an inflammatory and senescent phenotype.

## MATERIALS AND METHODS

### Isolation and culture of mouse SK-FBs

*Tfam^fl/fl^* UBC-Cre/ER^T2+/+^ and control *Tfam^fl/fl^* UBC-Cre/ER^T2−/−^ mice were used to obtain SK-FBs. *Tfam^fl/fl^* UBC-Cre/ER^T2+/+^ mice were obtained by mating *Tfam^fl/fl^* floxed mice (B6.Cg-*Tfam^tm1.1Ncdl^*/J) (Larsson et al., 1998) (The Jackson Laboratory, Bar Harbor, ME, USA) with UBC-Cre/ER^T2^ mice [B6.Cg-*Ndor1^Tg(UBC−cre/ERT2)1Ejb^*/1J] (Ruzankina et al., 2007) (The Jackson Laboratory). All animal procedures followed institutional guidelines and were approved by the Animal Experimentation Ethics Committee of the Centro de Biología Molecular Severo Ochoa (CBMSO), Universidad Autónoma de Madrid (UAM) (PROEX 005/18).

SK-FB cultures were established by explant growth of small fragments of tail skin in Dulbecco's modified Eagle's medium (DMEM) (Lonza, Verviers, Belgium) supplemented with 10% fetal bovine serum (HyClone, Cramlington, UK), 1 mM glutamine (Gibco, Paisley, UK), 1% penicillin/streptomycin (H317-603E, Lonza) and 55 µM β-mercaptoethanol (Gibco). Cultured cells were maintained at 37°C in 5% CO_2_, and the medium was replaced every 3 days. Cells were passaged when 90% confluence was reached using trypsin-EDTA (Gibco).

Cre/ER^T2^ expression was induced after treatment with 4-OHT (2.5 μM in ethanol) (Sigma-Aldrich, St Louis, MO, USA) for 6 days followed by consecutive doses of 1 μM 4-OHT every 6 days. Controls were treated with ethanol. SK-FB cultures treated with 4-OHT were also supplemented with 50 μg/ml uridine (Sigma-Aldrich) and 1 mM sodium pyruvate (Lonza), as previously described (King and Attardi, 1989).

### Analysis of *Cre* and *Tfam* genotype

Genomic DNA (gDNA) was extracted from cultured SK-FBs using an NZY Tissue gDNA Isolation kit (NZYTech, Lisboa, Portugal) according to the manufacturer's instructions. The presence of *Cre* gene, and *Tfam^fl/fl^* (WT) and *Tfam*^−/−^ (KO) genotype was determined by PCR. PCR conditions were as follows: initial denaturation at 95°C for 5 min, 35 cycles with denaturation at 94°C for 45 s, annealing at 58°C for 30 s and 45 s of extension at 72°C, and a final 10 min cycle of extension at 72°C. Primers used are listed in Table S1A. Amplicons were visualized in a 1% agarose gel using Gel Doc^TM^ XR+ and ChemiDoc^TM^ XRS+ Systems with Image Lab^TM^ Software (Bio-Rad Laboratories, Hercules, CA, USA).

### RNA extraction and quantitative real-time PCR (qRT-PCR) analysis

Total RNA was extracted using NZYol (NZYTech) following the manufacturer's protocol. One microgram of RNA was used for first-strand complementary DNA (cDNA) synthesis with a High-Capacity cDNA Transcription Kit (Applied Biosystems, Foster City, CA, USA). For qRT-PCR amplification, we used cDNA and primers added to Power Sybr Green PCR Master Mix (Applied Biosystems). Primer sequences are listed in Table S1B. *Hprt* gene was used as an endogenous reference. qRT-PCRs were performed on a Roche LightCycler^®^ 480 II Instrument (Roche Diagnostics, Mannheim, Germany). For relative quantification, we compared the amount of target normalized to the endogenous reference using 2^−ΔΔCt^ formula (Ct=threshold cycle).

### Western blot analysis

Cellular pellets were resuspended in lysis buffer [10 mM Tris-HCl pH 8.0, 150 mM NaCl, 0.1% sodium dodecyl sulfate (SDS), 1 mM EDTA pH 8.0] with a protease inhibitor (Roche Diagnostics). Protein concentration was determined by the DC method (Bio-Rad Laboratories) after sonication for 15 s. Samples were incubated for 5 min in 4× Laemmli sample buffer at 95°C. Cell lysates (15 µg) were electrophoresed by SDS-PAGE in 10% polyacrylamide gels (Bio-Rad Laboratories) and next transferred to nitrocellulose membranes (Bio-Rad Laboratories) by ultra-fast 3 min transference. After blocking for 1 h with skimmed milk in 0.1% TBS-Tween 20, membranes were incubated for 1 h at room temperature or overnight at 4°C with the following antibodies: rabbit polyclonal anti-mtTFA (1/250; ab47517, Abcam, Cambridge, UK) and anti-PGC-1α (1/500; sc13067, Santa Cruz Biotechnology, Santa Cruz, CA, USA), mouse monoclonal anti-ATPB (ATP synthase complex V β subunit) (1/1000; ab14730, Abcam), mouse monoclonal anti-VDAC1 (1/1000; ab14734, Abcam), anti-UQCRC2 (core protein 2 complex III) (1/2000; ab14745, Abcam) and anti-NDUFA9 (NADH:ubiquinone oxidoreductase subunit A9 complex I) (1/2000; ab14713, Abcam). Secondary antibodies were horseradish peroxidase (HRP)-conjugated goat anti-rabbit IgG (1/5000; G-21234, Invitrogen, Carlsbad, CA, USA) and goat anti-mouse IgG (1/5000; ab97023, Abcam).

Values were normalized to the β-actin levels that were detected using anti-β-actin HRP-conjugated antibody (1/25,000; ab49900, Abcam) as an internal reference. The protein bands were detected by chemiluminescence using Clarity^TM^ Western ECL Substrate (Bio-Rad Laboratories) in ImageQuant^TM^ LAS 400 (GE Healthcare, Buckinghamshire, UK) and quantified by densitometry using ImageJ software (http://rsb.info.nih.gov/ij).

### Analysis of mitochondrial supercomplexes and complex I in-gel activity assay

Mitochondrial-enriched fractions were isolated from SK-FBs grown in T175 flasks. Cellular pellets were washed twice in cold PBS and, after an incubation at −80°C for at least 15-30 min, were resuspended in a solution containing 20 mM Hepes-KOH pH 7.6, 220 mM mannitol, 70 mM sucrose, 1 mM EDTA and 0.5 mM PMSF. The extract was homogenized and centrifuged for 5 min at 4°C at 800 ***g***. Collected supernatants were centrifuged for 15 min at 4°C at 12,000 ***g*** and pellets (crude mitochondria) were resuspended again in previous solution and centrifuged for 15 min at 12,000 ***g*** at 4°C. Finally, the mitochondrial pellet was resuspended in sucrose buffer (10 mM Hepes pH 7.6 and 0.5 M sucrose) and quantified by a Pierce^TM^ MicroBCA kit (Thermo Fisher Scientific, Rockford, IL, USA). To prepare mitochondrial native proteins, 40-100 µg mitochondrial protein was solubilized in 100-200 µl of a buffer composed of 1.5 M aminocaproic acid, 50 mM Bis-Tris pH 7.0 and 4 g/g digitonin and incubated on ice for 15 min. After centrifugation for 30 min at 12,000 ***g*** at 4°C, the supernatant was combined with sample buffer (750 mM aminocaproic acid, 50 mM Bis-Tris, 0.5 mM EDTA, 0.02% SERVA Blue G-250) prior to loading (Moreno-Lastres et al., 2012).

Evaluation of the steady-state levels of the MRC enzymatic complexes was carried out by BNE, in 1D- or 2D-PAGE, and CI-IGA assay, following previously described protocols (Nijtmans et al., 2002). Proteins were transferred to nitrocellulose membranes, and the following antibodies were used for immunodetection: anti-UQCRC2 and anti-NDUFA9, and anti-COX5A (1/1000; ab110262, Abcam). Anti-SDHA (complex II subunit) (1/10,000; ab14715, Abcam) was used as mitochondrial protein reference. Secondary antibodies conjugated to HRP and band detection and quantification were used as aforementioned.

### Analysis of mitochondrial function and glycolytic activity

The OCR was used as an indicator of aerobic mitochondrial respiration and the ECAR as an indicator of glycolytic activity. Both OCR and ECAR were measured using a Seahorse XFp Analyzer (Agilent Technologies, Santa Clara, CA, USA). SK-FBs were seeded at 15,000 cells/well in a Seahorse XFp eight-well microplate (Agilent Technologies), and, after overnight incubation, two assays were performed. (1) Mito-Stress test: culture medium was replenished with XF Base Medium (Agilent Technologies) supplemented with 1 mM sodium pyruvate, 10 mM glucose (Sigma-Aldrich) and 2 mM glutamine, and microplates were incubated at 37°C under non-CO_2_ condition for 20 min. Mitochondrial function was assessed by injections of the following compounds: 1 μM oligomycin (Sigma-Aldrich), 0.6 μM FCCP (Abcam) and 1 μM rotenone/antimycin A (Sigma-Aldrich). Basal OCR, maximal respiration and ATP production (Divakaruni et al., 2014) have been determined by the Seahorse Wave Desktop Software (Agilent Technologies). (2) Glycolysis-stress test: culture medium was replenished with XF Base Medium supplemented with 2 mM glutamine, and cells were incubated for 20 min in a non-CO_2_ incubator. Glycolysis assay was performed upon SK-FB stimulation with sequential injections of 20 mM glucose, 1 μM oligomycin and 50 mM 2-deoxy D-glucose (Sigma-Aldrich). Glycolytic rates (TeSlaa and Teitell, 2014) have been determined by the Seahorse Wave Desktop Software.

### Detection of SA-β-gal activity

Quantification of senescence SK-FB in cultures was determined by SA-β-gal activity. The assay was performed as described in Debacq-Chainiaux et al. (2009). Cell nuclei were counterstained with 4′,6-diamidino-2-phenylindole (DAPI). Six random fields for each condition were photographed and digitalized by a Nikon Eclipse TE 2000-S microscope using the 100× objective. The total number of SK-FBs in the cultures was quantified by DAPI(+) nuclei using ImageJ software (http://rsb.info.nih.gov/ij), and the percentage of senescent SA-β-gal(+) SK-FBs was determined.

### Immunofluorescence of SK-FBs

SK-FBs grown on glass coverslips (25,000 cells) were immunolabelled with anti-α-SMA monoclonal antibody (1A4 clone, Sigma-Aldrich). Detection was performed with goat anti-mouse IgG_1_ Alexa Fluor 488-labelled antibody (Invitrogen) and DAPI counterstaining. Six random fields for each condition were photographed and digitalized using a Spot RT CCD camera and Spot 4.0.4 software (Diagnostic Instruments, Sterling Heights, MI, USA) on an Axioplan-2 fluorescence microscope (Zeiss, Jena, Germany) using a 200× objective. The total number of SK-FBs in the cultures was quantified by DAPI(+) nuclei using ImageJ software, and the percentage of α-SMA(+) cells was determined.

### Statistical analysis

Data were analysed using Prism software v6.0 (GraphPad Software, San Diego, CA, USA). Means were compared by Wilcoxon test, Mann–Whitney test and two-way ANOVA with Tukey's multiple comparison test, as appropriate. *P*<0.05 was considered statistically significant.

## Supplementary Material

Supplementary information
