# Supplementary material for: TFAM-deficient mouse skin fibroblasts – an ex vivo model of mitochondrial dysfunction
Source: Dis Model Mech. 2021 Aug 25;14(8):dmm048995. doi: 10.1242/dmm.048995 (PMC8405849; doi:10.1242/dmm.048995)
Supplement: Supplementary information [file dmm-14-048995-s1.pdf]

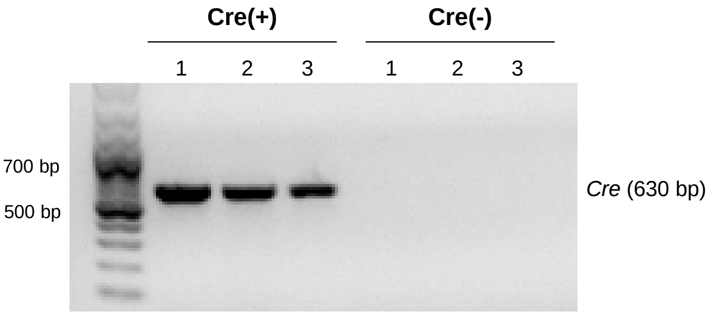

Fig. S1. Determination of *Cre* genotype by conventional PCR. Representative image of *Cre* amplification (630 bp) in 3 Cre(+) and 3 Cre(-) SK-FBs lines from different mice.

Disease Models & Mechanisms • Supplementary information

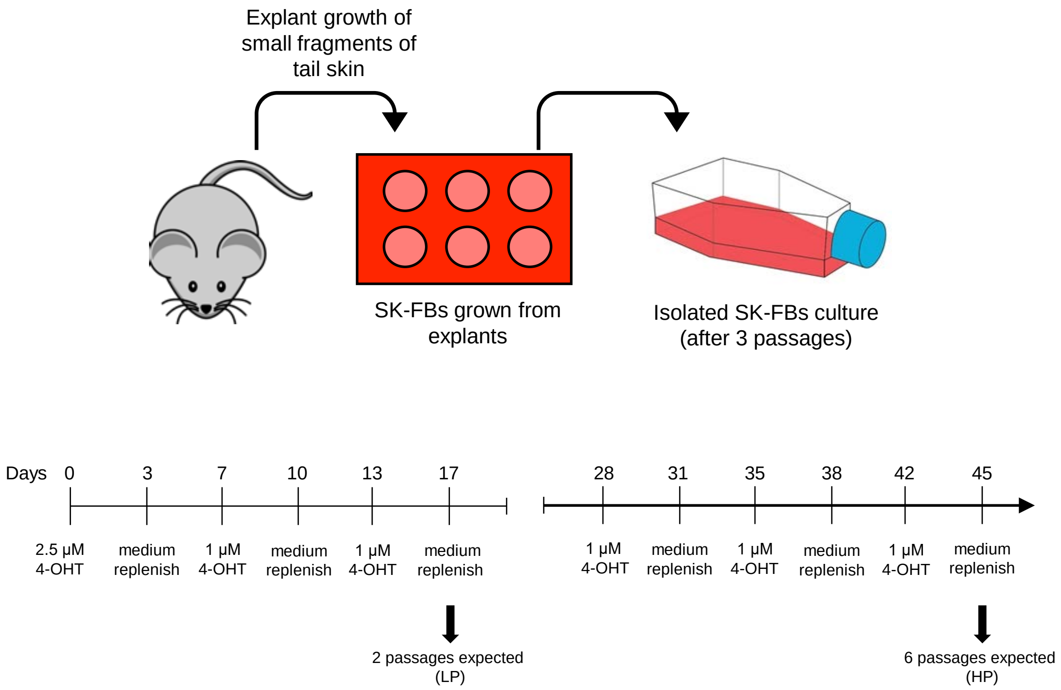

Fig. S2. Schematic representation of SK-FBs culture and treatment. The starting point (day 0) for the 4-OHT treatment should be considered after 3rd passage. Culture medium was replenished once between doses (each 3-4 days) with 4-OHT-free culture medium. Cells are predicted to be passaged with an average frequency of once per week.

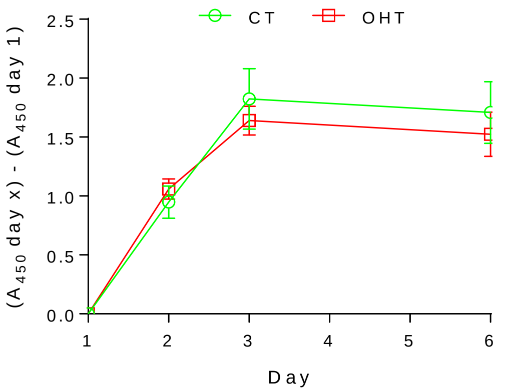

Fig. S3. Proliferation of Cre(+) SK-FBs. Treated (4-OHT) and untreated (CT) Cre(+) SK-FBs were seeded at 2500 cells/well in 96-well plates and proliferation was addressed by colorimetric detection of WST-1 at different time-points (n=6).

A

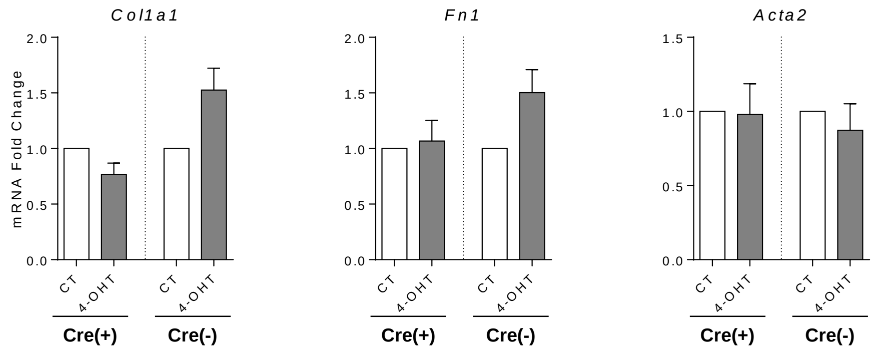

B

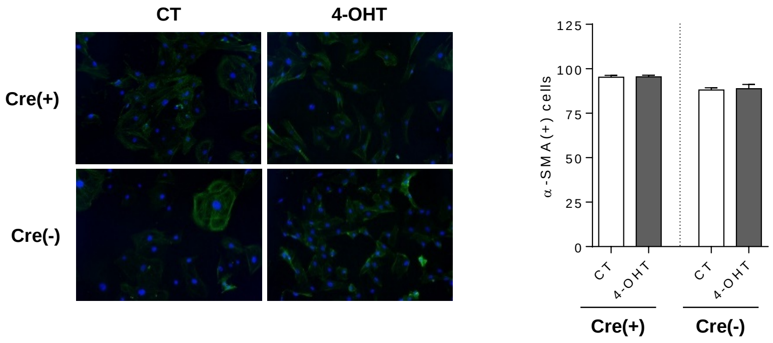

Fig. S4. Analysis of extracellular matrix components. (A) Changes in mRNA expression of treated (4-OHT) HP Cre(+) (n=9) and Cre(-) (n=4) cells with respect to untreated cells (CT) of extracellular matrix components *Col1a1*, *Fn1*, *Acta2.* **(B)** Immunofluorescent detection of a-SMA protein (green) counterstained with DAPI (blue) and quantification of α-SMA(+) 4-OHT and CT Cre(-) (n=2) and Cre(+) (n=2) SK-FBs (Mean±SEM).

Table S1. Primer sequences used for Genotyping *Cre* and *Tfam* **(A)** and qRT-PCR analysis of gene expression **(B)**.

A

Gene Name	Forward Primer Sequence	Reverse Primer Sequence
Tfam	5'- CTGCCTTCCTCTAGCCCGGG -3'	5'- GTAACAGCAGACAACTTGTG -3'<br>5’- CTCTGAAGCACATGGTCAAT -3’
Cre	5'- ATCCGAAAAGAAAACGTTGA -3'	5'- ATCCAGGTTACGGATATAGT -3'

Tfam: mitochondrial transcription factor A; Cre: cre-recombinase

B

Gene Name	Forward Primer Sequence	Reverse Primer Sequence
Hprt	5'- GTTGGGCTTACCTCACTGCT -3'	5'- TAATCACGACGCTGGGACTG -3'
Tfam	5’- CAGGAGGCAAAGGATGATTC -3’	5’-CCAAGACTTCATTTCATTGTCG-3’
mt-Co1	5’-CTCGCCTAATTTATTCCACTTCA-3’	5’-GGGGCTAGGGGTAGGGTTAT-3’
Sdhd	5’-CTTGAATCCCTGCTCTGTGG-3’	5’-AAAGCTGAGAGTGCCAAGAG-3’
Il6	5’-TAGTCCTTCCTACCCCAATTTCC-3’	5’TTGGTCCTTAGCCACTCCTTC-3’
Ccl2	5´-TTAAAAACCTGGATCGGAACCAA-3’	5’-GCATTAGCTTCAGATTTACGGGT-3’
Mmp3	5’-ACATGGAGACTTTGTCCCTTTTG-3’	5’-TTGGCTGAGTGGTAGAGTCCC-3’
Cxcl2	5’-CCAACCACCAGGCTACAGG-3’	5’-GCGTCACACTCAAGCTCTG-3’
Ifnb1	5’-CAGCTCCAAGAAAGGACGAAC-3’	5’-GGCAGTGTAACTCTTCTGCAT-3’
Col1a1	5’-CCTGAGTCAGCAGATTGAGAA-3’	5’-CCAGTACTCTCCGCTCTT-3’
Fn1	5´-ACCGACAGTGGTGTGGTCTA-3´	5´-CACCATAAGTCTGGGTCACG-3´
Acta2	5´-GTCCCAGACATCAGGGAGTAA-3´	5´-TCGGATACTTCAGCGTCAGGA-3´
Cdkn1a	5’-CCTGGTGATGTCCGACCTG-3’	5’-CCATGAGCGCATCGCAATC-3’
Cdkn2a	5’-CGCAGGTTCTTGGTCACTGT-3’	5’-TGTTCACGAAAGCCAGAGCG-3’

Hprt: hypoxanthine-guanine phosphoribosyltransferase; Tfam: mitochondrial transcription factor A; mt-Co1: mitochondrially encoded cytochrome C oxidase I; Sdhd: succinate dehydrogenase cytochrome b small subunit; Il6: interleukin 6; Ccl2: chemokine (C-C motif) ligand 2; Mmp3: matrix Metallopeptidase 3; Cxcl2: chemokine (C-X-C motif) ligand 2; Ifnb1: interferon beta 1; Col1a1: collagen type I alpha 1 chain; Fn1: fibronectin 1; Acta2: actin alpha 2; Cdkn1a: cyclin-dependent kinase inhibitor 1a; Cdkn2a: cyclin-dependent kinase inhibitor 2a

Disease Models & Mechanisms • Supplementary information
